# Effect of Nitrosative Stress on the *S*-Nitroso-Proteome of *Paracoccidioides brasiliensis*

**DOI:** 10.3389/fmicb.2020.01184

**Published:** 2020-06-04

**Authors:** Marina V. Navarro, Alison F. A. Chaves, Daniele G. Castilho, Isis Casula, Juliana C. P. Calado, Palloma M. Conceição, Leo K. Iwai, Beatriz F. de Castro, Wagner L. Batista

**Affiliations:** ^1^Department of Microbiology, Immunology and Parasitology, Escola Paulista de Medicina, Universidade Federal de São Paulo, São Paulo, Brazil; ^2^Department of Pharmaceutical Sciences, Instituto de Ciências Ambientais, Químicas e Farmacêuticas, Universidade Federal de São Paulo, Diadema, Brazil; ^3^Laboratory of Applied Toxinology, Center of Toxins, Immune-response and Cell Signaling, Instituto Butantan, São Paulo, Brazil

**Keywords:** *Paracoccidioides brasiliensis*, *S*-nitroso-proteome, nitric oxide, *S*-nitrosylation, nitrosative stress

## Abstract

The fungi *Paracoccidioides brasiliensis* and *Paracoccidioides lutzii* are the causative agents of paracoccidioidomycosis (PCM), a systemic mycosis endemic to Latin America. This fungus is considered a facultative intracellular pathogen that is able to survive and replicate inside macrophages. The survival of the fungus during infection depends on its adaptability to various conditions, such as nitrosative/oxidative stress produced by the host immune cells, particularly alveolar macrophages. Currently, there is little knowledge about the *Paracoccidioides* spp. signaling pathways involved in the fungus evasion mechanism of the host defense response. However, it is known that some of these pathways are triggered by reactive oxygen species and reactive nitrogen species (ROS/RNS) produced by host cells. Considering that the effects of NO (nitric oxide) on pathogens are concentration dependent, such effects could alter the redox state of cysteine residues by influencing (activating or inhibiting) a variety of protein functions, notably *S*-nitrosylation, a highly important NO-dependent posttranslational modification that regulates cellular functions and signaling pathways. It has been demonstrated by our group that *P. brasiliensis* yeast cells proliferate when exposed to low NO concentrations. Thus, this work investigated the modulation profile of *S*-nitrosylated proteins of *P. brasiliensis*, as well as identifying *S*-nitrosylation sites after treatment with RNS. Through mass spectrometry analysis (LC-MS/MS) and label-free quantification, it was possible to identify 474 proteins in the *S*-nitrosylated proteome study. With this approach, we observed that proteins treated with NO at low concentrations presented a proliferative response pattern, with several proteins involved in cellular cycle regulation and growth being activated. These proteins appear to play important roles in fungal virulence. On the other hand, fungus stimulated by high NO concentrations exhibited a survival response pattern. Among these *S*-nitrosylated proteins we identified several potential molecular targets for fungal disease therapy, including cell wall integrity (CWI) pathway, amino acid and folic acid metabolisms. In addition, we detected that the transnitrosylation/denitrosylation redox signaling are preserved in this fungus. Finally, this work may help to uncover the beneficial and antifungal properties of NO in the *P. brasiliensis* and point to useful targets for the development of antifungal drugs.

## Introduction

Paracoccidioidomycosis (PCM) is a granulomatous systemic disease caused by fungi of the *Paracoccidioides* genus. PCM is restricted to Latin America and has a significant number of reported cases in Brazil (Martinez, 2017) with high prevalence and mortality rates in the South, Southeast and Midwest regions (Almeida et al., 2017) affecting mainly individuals involved in agricultural activities. The disease has several clinical presentations, with manifestations ranging from the lungs to the skin, and is severe for immunocompromised patients (Shikanai-Yasuda et al., 2018).

*Paracoccidioides* spp. are thermally dimorphic fungi existing as mycelia in the environment, and when inhaled, the mammalian body temperature (37°C) induces its transition to yeast form. Pulmonary resident macrophages recognize fungal cell wall pathogen-associated molecular patterns (PAMPs) and have mechanisms to eliminate these pathogens, such as phagocytosis and the production of reactive oxygen species and reactive nitrogen species (ROS/RNS) (de Castro et al., 2018). Oxidative and nitrosative stress are disorders caused by increases in ROS and RNS levels. Usually, ROS levels are maintained at baseline levels in aerobic organisms but are constantly produced during respiration. Moreover, ROS are produced by oxidase enzymes, which are essential to the immune system response against a pathogen. Proteomic studies in *P. lutzii* have demonstrated that various proteins involved in oxidative stress are differentially expressed according to different concentrations of H_2_O_2_ exposure (de Arruda et al., 2013), and different concentrations of H_2_O_2_ lead to differentiated patterns of phosphorylation in *P. brasiliensis*, which determines different responses, that is, survival or cell death (Chaves et al., 2017).

Similarly, nitric oxide (NO) is produced by macrophages and is associated with the transition of mycelia to yeast inhibition, which is highly important during infection (Gonzalez et al., 2007). Previous studies with *P. lutzii* demonstrated that treatment with a NO donor leads to a reduction in the mitochondrial electron transport chain due to nitrosative stress, as well as an increased expression of superoxide dismutase (SOD) and cytochrome c peroxidase (CCP), which is also associated with oxidative stress (Parente et al., 2015). NO can react with a great variety of targets, both outside and inside cells. NO may activate and inhibit enzymes, ion channels or transcription factors (Maniscalco et al., 2016). Alteration of cysteine residue redox status is an established event and can influence several protein functions. There are many oxidative reactions, such as sulfonic acid formation or reversible modifications, such as sulfenic and sulfinic acid, glutathionylation, disulfide formation and *S*-nitrosylation (Spickett et al., 2006).

*S-*nitrosylation, such as phosphorylation, is a reversible post-translation modification (PTM) that occurs in a cysteine (Cys) which is converted to a nitrosothiol. This NO-dependent PTM has been shown to regulate a considerable variety of cell signaling events and consequently different cell functions (Batista et al., 2013; Haniu et al., 2013; Conceição et al., 2019; Fernando et al., 2019). *S*-nitrosylation can generate important conformational changes in the protein, which can lead modifications in the protein-protein interaction, allowing other PTMs to occur, such as phosphorylation, acetylation, ubiquitination, and disulfide bond formation (Hess and Stamler, 2012; Selvakumar et al., 2013; Fernando et al., 2019). Thus, protein *S*-nitrosylation can provide the basis for physiological regulation based on modifications of cellular redox status (Stamler et al., 2001; Hess et al., 2005; Lillo et al., 2019; Ma et al., 2020).

In fungi, ROS/RNS are known to be cytotoxic (Calich et al., 2008), however, a previous study by our group demonstrated the ability of *P. brasiliensis* to proliferate under low concentrations of H_2_O_2_ and NO (Haniu et al., 2013; Conceição et al., 2019). These data suggest that the fungus can benefit from low concentrations of ROS and RNS to survive and proliferate. Knowing that these events are also regulated by redox PTMs, we used a proteomic strategy associated with biotin-switch technique (BST) to identify the *S*-nitrosylated proteins in *P. brasiliensis* treated with different concentrations of NO.

## Materials and Methods

### Fungus Isolate and Growth Conditions

*Paracoccidioides brasiliensis* (isolate Pb18) was used in all experiments. This isolate was cultivated on yeast extract peptone dextrose modified medium (mYPD) (0.5% w/v yeast extract, 1.0% w/v peptone and 0.5% w/v glucose, and 1.4% w/v agar, pH 6.5 or 5.5) at 37°C for the growth of the *P. brasiliensis* yeast phase.

### Growth Assay

Yeast cells were cultivated in mYPD broth pH 6.5 at 37°C under constant shaking (150 rpm) for 5–7 days. Next, the cells were washed with PBS (pH 7.2) and seeded (1.8 × 10^5^) in a 6-well culture plate with F12 medium for 24 h. Then, cells were washed with PBS (pH 7.2) resuspended in mYPD and exposed a range of 0.25–1,000 μM of sodium nitrite at pH 5.5 (NaNO_2_ release NO in middle acid medium) or *S*-nitrosoglutathione (GSNO) produced in-house (Sahoo et al., 2006) at 37°C under shaking. After stimulation, yeasts were disaggregated by passing 5-times in a 1 mL syringe and 21-gauge needle. Then, the yeast suspension was applied in a cell strainer (Becton, Dickinson and Company – Franklin Lakes, NJ, United States). This procedure allowed the disassociation of the yeast aggregates (characteristic in *Paracoccidioides* spp. cultures) facilitating the counting of individual yeasts. Cell viability was determined by Trypan Blue using Neubauer chamber for 4 and 8 days. We also evaluated the cell proliferation by colony formation unit (CFU) counts. After NO treatment (0.25 or 10 μM), yeast cell suspension aliquots (100 μL) were plated in BHI plates supplemented with Fetal Bovine Serum (FBS) for 7 days at 37°C. The experiment was repeated three times.

### NO Treatment and Lysate Preparation

Yeast cells were grown in mYPD pH 6.5 at 37°C and 150 rpm for 5–7 days. Yeast cells were centrifuged at 3,000 × *g* for 15 min, and the supernatant was discarded. The pellet was washed with PBS (pH 7.2), resuspended in defined Ham’s F12 medium and cultivated at 37°C under constant shaking (150 rpm) for 24 h. Next, the cells were centrifuged at 3,000 × *g* for 15 min, washed twice in PBS (pH 7.2) and resuspended in mYPD pH 5.5. Different concentrations of NaNO_2_ (0, 25, and 10 μM) were added to yeast cells (in slightly acidified medium, NaNO_2_ releases NO according to the literature (Schnappinger et al., 2003; Rhee et al., 2005) and incubated for 5 h at 37°C with constant shaking (180 rpm) protected from light.

Yeast cells were washed and resuspended in pre-cold lysis buffer (50 mM Tris, 500 mM NaCl, 0.1% EDTA, 1% NP-40, 1 mM PMSF and 10 mM N-ethylmaleimide) with protease inhibitors (cOmplete^TM^ Protease Inhibitor Cocktail, Roche Diagnostics GmbH Mannheim, Germany). Glass beads were added (425–600 μM Sigma-Aldrich, St. Louis, MO, United States), and samples were vortexed to disrupt yeast cells for 5 cycles of 90 s (Villén et al., 2008). Cellular debris was separated by centrifugation (3,000 × *g*, 15 min). Protein concentration was measured using Bradford Coomassie reagent and bovine serum albumin was used as standards (Bradford, 1976).

### Immunoblotting Analysis

Total cell lysates (50 μg/mL) were subjected to electrophoresis on 10% SDS-polyacrylamide gels and transferred onto nitrocellulose membranes (Thermo Fisher Scientific, Waltham, MA, United States). Blots were probed using specific monoclonal antibodies against Nitroso-Cys (Ag Scientific) and Nitro-Tyr (Cell Signaling) at a 1:1,000 dilution. After incubation with the appropriate HRP-conjugated secondary antibodies (at 1:2,000 dilution), blots were developed using the Super Signal system (Thermo-Pierce – Rockford, IL, United States). Image acquisition and densitometry were carried out using a chemiluminescence documentation system (UVITEC, Cambridge, United Kingdom).

### Biotin-Switch Assay and Protein Identification

The biotin-switch assay was performed according to protocol (Forrester et al., 2009; Chen et al., 2010), with modifications. Briefly, 2 mg of protein was mixed with 2 volumes of HEN buffer (100 mM HEPES, 1 mM EDTA, 0.1 mM Neocuproine, pH 8.0) followed by the addition of SDS (25% v/v) and 1 M iodoacetamide (IAA) to final concentrations of 2.5% and 20 mM, respectively. Following frequent vortexing at 50°C for 20 min, proteins were precipitated with 3 volumes of acetone at –20°C for 20 min. Proteins were recovered by centrifugation at 5,000 × g for 5 min, and the pellet was gently washed 4 times with 1 mL 70% acetone/H_2_O. The pellet was resuspended in 240 μL of HENS buffer (HEN buffer containing 1% SDS). The samples were mixed with 0.1 volume of biotin-HPDP (2.5 mg/mL – Sigma-Aldrich) and 0.1 volume of freshly prepared ascorbate (200 mM) in HEN buffer following incubation at room temperature and orbital stirring for 1 h. All reactions were performed in the dark.

Samples were acetone-precipitated and after washing, the pellet was resuspended in 250 μL of HENS/10 (HENS diluted 10-fold into H_2_O) followed by the addition of 750 μL of neutralization buffer (25 mM HEPES, 100 mM NaCl, 1 mM EDTA, pH 7.5). Samples were incubated at 4°C for 16 h with 40 μL of streptavidin-agarose beads. The beads were washed 4 times with 1 mL of wash buffer (neutralization buffer plus 600 mM NaCl). Samples were eluted with 20 μL of elution buffer (guanidine hydrochloride 8 M).

### Protein Reduction, Alkylation, Digestion, and Peptide Desalting

Enriched proteins were denatured with 4 M guanidine hydrochloride (GuHCl), and disulfide bonds were reduced with 5 mM 1,4-dithiothreitol (DTT) at 65°C for 1 h. Then, 15 mM IAA was added and incubated at room temperature (in the dark) for 1 h. Samples were incubated with 10 mM DTT for 15 min at room temperature, and proteins were precipitated with 8 volumes of cold acetone and 1 volume of cold methanol for 3 h at -80°C and centrifuged at 14,000 × *g* for 10 min at 4°C. The supernatant was discarded, and the precipitate was washed (2 times) with cold methanol and dried in a SpeedVac (Savant SpeedVac, Thermo Fisher Scientific Inc., Asheville, NC, United States). Proteins were dissolved in 10 μL 100 mM NaOH, 15 μL H_2_O_2_ and 75 μL of 50 mM HEPES buffer (pH 7.5); subsequently, 2 μg of sequencing-grade modified trypsin (Promega, Madison, WI, United States) was added to samples and incubated overnight at 37°C (Kleifeld et al., 2011; Castilho et al., 2014). Samples were diluted up to 500 μL of 0.1% trifluoroacetic acid (TFA) for trypsin inactivation and loaded into a solid-phase extraction Sep-Pak C18 cartridge (Waters, Milford, MA, United States) according to the protocol (Menin et al., 2008). After desalting, samples were dried in SpeedVac and resuspended in 0.1% formic acid prior to nanoliquid chromatography-tandem mass spectrometry (LC-MS/MS) analysis.

### Mass Spectrometry Analysis

Enriched samples were analyzed using an LTQ-Orbitrap Velos mass spectrometer (Thermo Fisher Scientific, Waltham MA, United States) connected to an Easy-nLCII (Thermo Fisher Scientific, Bremen, GA, United States) operating in positive ionization mode with voltage 2.3 kVe and temperature at 250°C. Peptides were separated with a 90 min gradient elution, with 5–40% solvent B (0.1% formic acid in acetonitrile) followed by a 20 min gradient with 40–95% solvent B at a flow rate of 200 nL/min. Columns were packed in house where the precolumn (ID 100 μm × OD 360) was packed with 5 cm of C18 resin (10 μm, Phenomenex Inc, Torrance, CA, United States) and analytical column (ID 75 μm × OD 360) was packed with 10 cm of ACQUA C18 resin (5 μm, Phenomenex Inc, Torrance, CA, United States). The mass spectrometer was programmed in the data-dependent acquisition mode with a resolution of 30,000 (400 m/z) followed by collision induced by dissociation (CID) of the 20 most intense ions. A dynamic exclusion time of 70 s was employed. The isolation window for the precursor ions was set to 2 Da, and the minimum number of ions to trigger events MS2 was 5000. The analyses were run in triplicate.

### Data Analysis

The MS raw files were processed and searched using MaxQuant version 1.5.2.8 (MPI in Biochemistry, Martinsried, Germany) software integrated into the Andromeda Search engine. Proteins were identified by querying against the full sequence of *P. brasiliensis* strain *Pb18* (Proteome ID: UP000001628, UniProt)^1^. Searches were performed considering carbamidomethylation of Cys as a fixed modification and oxidation of Met and phosphorylation in Ser, Thr, or Tyr as variable modifications. All validated proteins were submitted to Gene Ontology (GO), Blast (*e*-value ≤ 1e-5) and InterProannotation using the Blast2GO algorithm (Conesa and Götz, 2008). Only proteins identified in at least two analyses were used for validation and table assembly. The mass spectrometry proteomics data have been deposited to the ProteomeXchange Consortium via the PRIDE (Perez-Riverol et al., 2019) partner repository with the dataset identifier PXD018775.

### Enzymatic Activity Assay

The activity of Cu-Zn-superoxide dismutase (SOD) was analyzed colorimetrically at 560-nm wavelength by measuring the reduction of nitroblue tetrazolium (NBT) by superoxide radicals, which were generated from PMS reduction mediated by β-NADH, where 1 unit is the SOD amount necessary to inhibit 50% of the initial rate of NBT reduction (Flohé et al., 1984; Ewing and Janero, 1995; Castilho et al., 2018). The activity of catalase (CAT) was determined by monitoring the decrease in absorbance at 240 nm wavelength of the rate of hydrogen peroxide decomposition (Aebi, 1984). One unit of CAT activity is equivalent to the amount of enzyme that decomposes 1 mmol of H_2_O_2_ per 1 min in pH 4.5 at 25°C.

The activity of glutathione peroxidase (GPx) is based on the ability of enzyme to catalyze oxidation of organic peroxides in the presence of reduced NADPH, and then the samples were analyzed colorimetrically at 340 nm wavelength (Wendel, 1981; Flohé and Günzler, 1984). One unit of GPx activity was represented as the amount of enzyme catalyzing the oxidation of 1 μmol of NADPH/min under assay conditions and is expressed as U/mg of protein. The activity of glutathione reductase (GR) was determined colorimetrically at 340-nm wavelength by measuring NADPH consumption rate, since GR catalyzes the NADPH-dependent reduction of oxidized glutathione (GSSG) to reduced glutathione (GSH) (Carlberg and Mannervik, 1975). The oxidation of 1 μmol of NADPH/min in pH 7.6 at 25°C was used as a unit of GR activity and is expressed as U/mg of protein. The absorbance was measured on a SpectraMax^®^ Plus 384 spectrophotometer (Molecular Devices, San Jose, CA, United States).

### Quantitative Determination of GSH and GSSG

Yeast cells were lysed with glass beads in buffer containing 50 mM Tris-HCl [pH 7.5], 2 mM EDTA, 50 mM KCl, 0.2% Triton X-100, 1 mM sodium orthovanadate, 1 mM phenylmethylsulfonyl fluoride and protease inhibitor (Roche Diagnostics GmbH Mannheim, Germany) (Villén et al., 2008). GSH and GSSG concentrations were determined largely as described in the literature (Rahman et al., 2007). After the detection of GSH and GSSG values, the GSH/GSSG ratio was calculated and the profile of the cellular redox environment was defined.

### Statistical Analysis

The techniques performed in this study were validated with the reproducibility of three independent experiments. Variance analysis was performed followed by Student’s *t*-test, with *p* < 0.05 considered to be significant.

## Results

### *P. brasiliensis* Response to Nitrosative Stress

**FIGURE 1:**
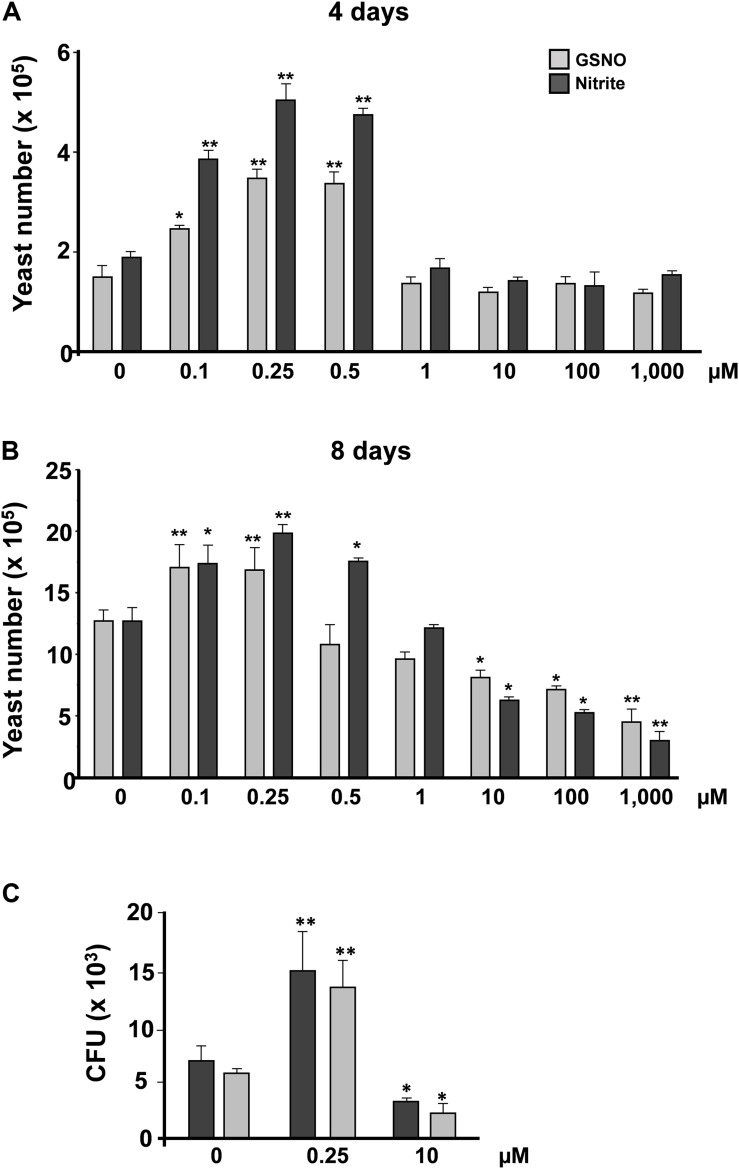
*P. brasiliensis* yeast counting after treatment with reactive nitrogen species. Yeast cells of *P. brasiliensis* were treated with varying concentrations of reactive nitrogen species. The cells were incubated for **(A)** 4 and **(B)** 8 days at 37°C (*n* = 3 each point). Next, the cells were counted in Neubauer chamber. The graph shows the mean ± SD of each sample. **(C)** Treated yeast cells were plated in BHI and incubated at 37°C for 7–10 days (*n* = 6 at each point). The graph shows the means ± SD of total CFU before and after treatment with nitrite or GSNO for each concentration. This result is representative of three independent experiments with *p <0.05 and **p <0.01 (comparison with no treatment control).

Previous studies by our group have demonstrated that *P. brasiliensis* presents distinct responses when challenged with different concentrations of ROS and RNS (Haniu et al., 2013; Chaves et al., 2017). Low concentrations of ROS and RNS induced a proliferative response; in contrast, with higher doses, a survival pattern is observed. However, it is not clear whether this response remains in the presence of different NO donors. Hence, we tested the response of *P. brasiliensis* after incubation with *S*-nitrosoglutathione (GSNO) and sodium nitrite (pH 5.6). Yeast cells were cultivated for 5 days in mYPD at 37°C with varying concentrations of these two compounds (0.1–1,000 μM). After 4 days of treatment, we observed that both nitrite and GSNO induced a proliferative response at 0.1–0.5 μM (Figure 1A). In contrast, 1, 10, 100, and 1,000 μM of both compounds did not present significant differences in cell viability. After 8 days, we observed the same results at low NO concentrations, but higher concentrations induced cell death in a dose-dependent manner (Figure 1B). These profiles of cell growth and death were confirmed by counting colony-forming units (CFU). Yeast cells treated with low concentrations of nitrite or GSNO (0.25 μM) responded with significant cell proliferation (13.7 ± 2.4 × 10^3^ and 15.2 ± 3.9 × 10^3^ CFU, respectively) compared to control (5.8 ± 0.45 × 10^3^and 6.9 ± 1.3 × 10^3^ CFU, respectively) (Figure 1C). On the other hand, high concentrations of nitrite and SNOG (10 μM) induced a reduction in cell viability (2.4 ± 0.81 × 10^3^ and 3.4 ± 0.2 × 10^3^ CFU, respectively) when compared to control. These data demonstrate that GSNO and nitrite (under pH 5.6) induce similar responses in the exposure of *P. brasiliensis* yeast cells, allowing fungal proliferation at low levels of NO or a survival response at high concentrations of RNS. Being a radical, NO (^⋅^NO) is also involved in both anti- and pro-oxidant mechanisms. As an antioxidant, NO can modify cellular processes to confer protection to cells against oxidative damage (Wink et al., 2001; Fernando et al., 2019). Conversely, as a pro-oxidant, NO could react with molecular oxygen or ROS (especially superoxide: O_2_^⋅⁣–^) and be converted to a strong oxidant, reactive nitrogen oxide species (RNOS, especially peroxynitrite: ONOO^–^), that could oxidatively damage a variety of biological molecules.

### *S*-Nitrosylation and Nitration Profile of *P. brasiliensis* After Nitrosative Stress

Fundamental processes, such as cell growth, adaptation, survival and differentiation, are guided by PTMs. Among PTMs, phosphorylation in serine-, threonine-, and tyrosine-specific residues is the most common, however, a considerable number of experimental studies regarding free radical participation in physiological and pathophysiological processes have emerged (Hess et al., 2005; Arai et al., 2008; Tsujita et al., 2008; Maniscalco et al., 2016). Protein oxidative modifications can alter protein activity in a reversible way and is considered a regulating mechanism (Spickett et al., 2006; Monteiro et al., 2008). The most understood and widely reported of these modifications is *S*-nitrosylation, a reversible PTM that occurs when there is a covalent bound of a nitrogen monoxide group to the free thiol group belonging to the side chain of amino acid cysteine (Cys). It has been shown that posttranslational modification introduced into Cys residues from various classes of proteins may be important for regulating protein function. Thus, protein *S*-nitrosylation could provide the basis for a mechanism of physiological regulation based on intracellular redox status changes (Stamler et al., 2001; Hess et al., 2005).

**FIGURE 2:**
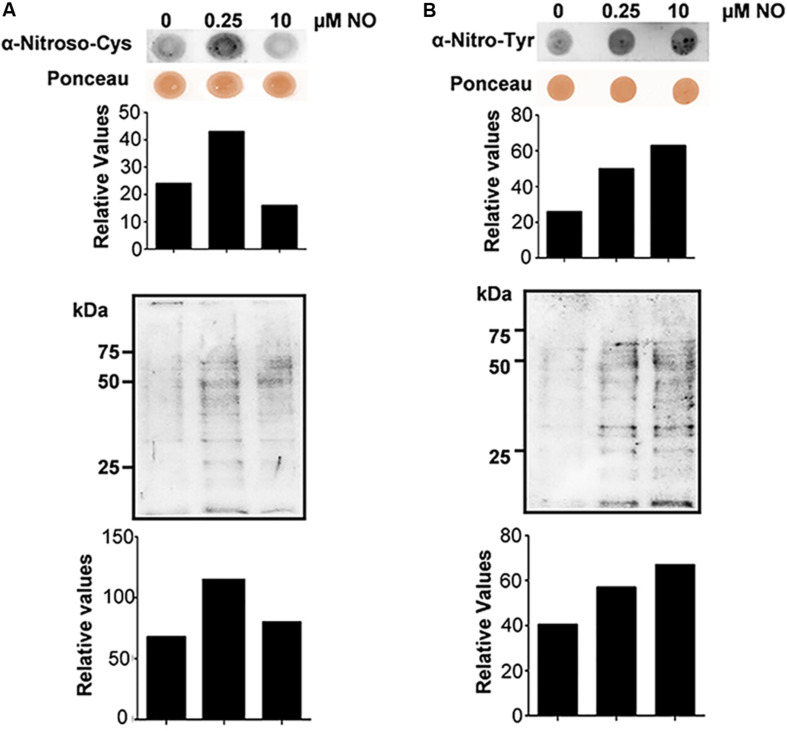
*S*-nitrosylation and nitration profile of *P. brasiliensis* proteins before and after treatment with NO. Cells were subjected to lysis and the protein extracts were analyzed by Dot blotting and Western blotting with anti-nitroso-Cys **(A)** or anti-nitro-Tyr **(B)** antibody. The graphs were obtained by densitometric analysis of the bands.

In this context, we evaluated the *S*-nitrosylation profile of *P. brasiliensis* proteins after treatment with nitrite (pH 5.6). Thus, dot blot and western blot assays were performed with anti-nitroso-Cys antibodies (1:1,000). In Figure 2A, it is possible to observe differences in the profile of *S*-nitrosylation among the tested extracts. There was an approximately twofold increase in the labeling of proteins obtained from cells treated with 0.25 μM NO compared to the control without treatment (Figure 2A, upper panel). At 10 μM nitrite, no significant labeling variations of *S*-nitrosylated proteins were observed (Figure 2A, upper panel) compared to control. A similar profile was observed in samples tested by western blot analysis (Figure 2A, lower panel). This result suggests that *S*-nitrosylation regulation in *P. brasiliensis* is not dose-dependent, presenting a more complex regulation. Due to the reversible character of this PTM, it is possible to say that high concentrations of nitrite induced the stress response machinery, thereby reducing the *S*-nitrosylation levels of the proteins. In addition to *S*-nitrosylation, another important redox posttranslational modification is protein nitration, which is an irreversible posttranslational modification that occurs by the action of peroxynitrite on tyrosine residues. Nitration may interfere directly or indirectly with protein phosphorylation/dephosphorylation and, consequently, with cell signaling pathways (Monteiro et al., 2008).

Considering the importance of protein oxidation in intracellular signaling pathway regulation, we analyzed the nitration levels of proteins in *P. brasiliensis* subjected to nitrosative stress. Thus, dot blot and western blot assays were performed with anti-Nitro-Tyr antibody (1:2,000). In Figure 2B, it is observed that both low (0.25 μM) and high (10 μM) nitrite concentrations were able to induce an increase in fungal protein nitration levels when compared to the sample control. In both assays, the increase in labeling in both the dot blot (Figure 2B, upper panel) and western blot (Figure 2B, lower panel) was dose dependent, and at 10 μM nitrite (pH 5.6), higher protein labeling was observed with a consequent increase in nitration levels of Tyr. Protein nitration, as previously mentioned, is a PTM that relies on peroxynitrite formation through superoxide anion and NO reactions. Peroxynitrite can damage a wide variety of molecules in cells, including lipid peroxidation, tyrosine nitration, thiols, amines and fatty acids and hydroxylate guanine nucleotides at acidic pH (Ferrer-Sueta et al., 2018). In particular, tyrosine-nitrated proteins are extremely useful as indicators of oxidative/nitrosative stress and inhibit several antioxidant enzymes, such as superoxide dismutase, which can lead to cellular stress (Ischiropoulos et al., 1992; Ahmad et al., 2019).

### Redox Status Evaluation of *P. brasiliensis* After Nitrosative Stress

Glutathione (GSH) coupling with the enzyme glutathione reductase (GR) is one of the most important cellular antioxidant systems. GSH (reduced form) is able to eliminate both ROS and RNS, consequently controlling redox homeostasis. These antioxidants systems also participate in the maintenance ROS and RNS at a nontoxic concentration to protect microorganisms. Under oxidative stress conditions, GSH is oxidized to GSSG; thus, the GSH:GSSG ratio is altered. Therefore, the dosage of GSH and GSSG and the evaluation of antioxidant enzyme activity (glutathione peroxidase, glutathione reductase, superoxide dismutase and catalase) reflect individual stress conditions.

**FIGURE 3:**
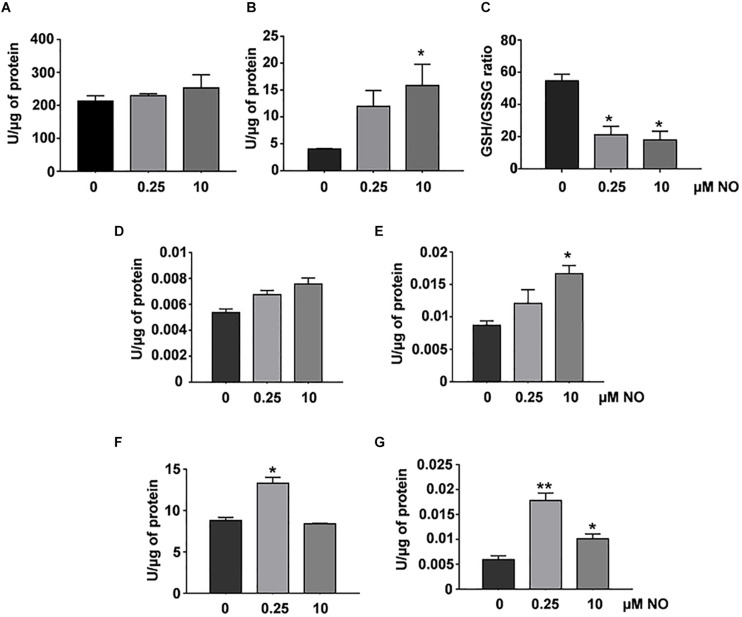
Redox status and antioxidant enzymatic activity in *P. brasiliensis* treated with different concentrations of NO. GSH **(A)** and GSSG levels **(B)** were run as described in section “Materials and Methods.” The GSH/GSSG ratios **(C)** are the GSH levels divided by the GSSG levels. Enzymatic activities of GPx **(D)**, GR **(E)**, SOD **(F)**, or catalase **(G)** were assessed in total protein extract of the fungus treated with varying concentrations of NO. The results are expressed as specific activity (units of enzyme/μg protein). All the data shown in this figure were analyzed using Student’s *t*-test. Error bars correspond to the standard deviations of the measurements made in triplicate. *p < 0.05 and **p < 0.01 (comparison with no treatment control).

Initially, we evaluated the concentration of reduced (GSH) and oxidized (GSSG) glutathione, as well as the activity evaluation of glutathione reductase and glutathione peroxidase enzymes. No changes were observed in the reduced GSH levels of *P. brasiliensis* after treatment with different concentrations of nitrite (Figure 3A). On the other hand, in Figure 3B, an increase in GSSG levels (oxidized glutathione) was observed in fungi treated with 10 μM nitrite. The GSH:GSSG (reduced glutathione: oxidized glutathione) ratio is an important marker of oxidative stress, and the ratio between GSH and GSSG must be very high for the maintaining of intracellular reducing power (Kappus, 1987). After treatment with different concentrations of nitrite, a decrease in the GSH/GSSG ratio was observed in both conditions (Figure 3C). All these data demonstrate that both low and high NO concentrations can modulate the intracellular redox status in *P. brasiliensis*.

The glutathione peroxidase (GPx) activity did not show significant variations among the evaluated samples, however, we observed an increasing tendency in its activity after treatment with 10 μM nitrite (Figure 3D). The GR activity showed a significant increase after stimulation with high NO concentrations (Figure 3E). We observed an increase SOD activity after treatment with low NO concentration. At high concentration, there was not statistical significance in relation to control (Figure 3F). The absence in SOD activity may be related to the increase in nitration levels (Figure 2B), a well-known indirect marker of the presence of peroxynitrite, which is associated with decreased SOD activity (Demicheli et al., 2018). Interestingly, catalase activity increased (∼3-fold) after stimulation with low NO concentrations, whereas at the concentration of 10 μM NO, a 1.7-fold increase was observed (Figure 3G). These data showed that RNS concentration can differentially modulate the response of *P. brasiliensis* to stress caused by NO. Such enzymes as catalase and SOD are considered the first line of metalloenzymes related to oxidative stress protection (Davidson et al., 1996).

### *S*-Nitroso-Proteome of *P. brasiliensis* Under Nitrosative Stress

After evidencing the existence of a redox imbalance and variation in redox PTM (*S*-nitrosylation and nitration) levels of *P. brasiliensis* yeast cells under nitrosative stress, we performed a proteomic analysis to identify *S*-nitrosylation sites in proteins from *P. brasiliensis*. We used the biotin-switch technique (BST) methodology (Forrester et al., 2009); this assay has become critical for mapping of *S*-nitrosylated proteins in different biological models. This technique consists of three main steps: (1) free thiols are blocked with methylmethane tetrisulfonate (MMTS, a reactive thiosulfonate); (2) SNOs is decomposed and reduced to free thiol by transnitrosation with ascorbate; and (3) labeled with a thiol-specific biotinylation reagent (biotin-HPDP), a mixed biotin-reactive disulfide. Western blot analysis was performed to ensure that the proteins identified were actually *S*-nitrosylated and demonstrated reaction specificity. Control groups used were absent of MMTS blocking or biotin. No unspecific labeling was observed (data not shown).

*S*-nitrosylated proteins were enriched by streptavidin-based affinity chromatography, selectively eluted with β-mercaptoethanol and identified in the nLC-MS/MS system on Orbitrap-Velos equipment (Thermo Fisher Scientific, Waltham, MA, United States). The results were analyzed using the high-energy electron fragmentation method for collision (CID). The generated files (.raw) were submitted to the MaxQuant search algorithm. The resulting spectra were analyzed in the MaxQuant search software (version 1.5.2.8). Overall, 474 *S*-nitrosylated proteins were identified, of which 196 were identified in the control group, 138 were identified in the group treated with 0.25 μM NO and 140 proteins in the group treated with high NO concentrations. The distribution of the mass error was near zero, and most values were less than 1 pep (posterior error probability) (Supplementary Figure S1A), these values satisfactorily guarantee the mass accuracy of MS data. Furthermore, peptides size varied between 7 and 44 amino acids (Supplementary Figure S1B), corresponding a typical tryptic digestion, confirming that sample preparation reached the technical standard.

**FIGURE 4:**
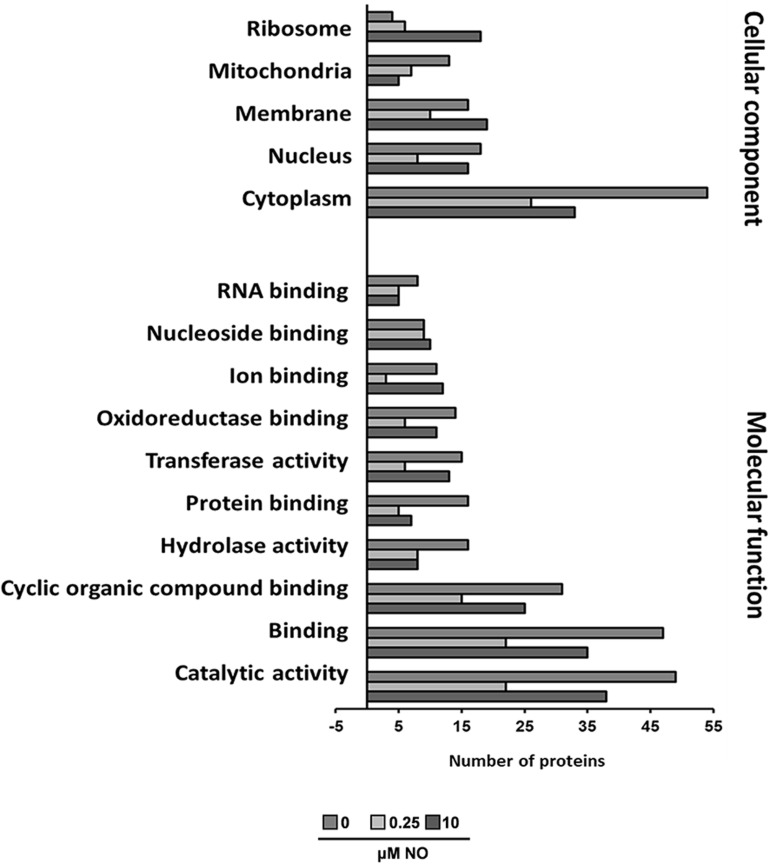
Functional categorization of Blast2GO. The lists of *P. brasiliensis* proteins identified by mass spectrometry were analyzed using Blast2GO for molecular function and cellular component.

To estimate the nature and functionality of the *S*-nitrosylated proteins identified, we performed Blast2GO analyses. For Blast2GO enrichment, 474 *S*-nitrosylated proteins were enriched in cellular component and molecular function (Figure 4). In general, most of the functions are related to catalytic activity, binding proteins and enzymatic activity (oxidoreductases, transferases, and hydrolases), and most of the proteins are found in the cytoplasmic region (Figure 4). The presence of *S*-nitrosylated proteins in the control group indicates that *P. brasiliensis* produces endogenously RNS. Different fungi have shown the ability to produce NO (Zhao et al., 2015; Pengkit et al., 2016). Despite the discovery of Nitric Oxide Synthase (NOS-like) in several fungi, it is not clear how endogenous NO production occurs in fungi (Pengkit et al., 2016). This way, it is possible that the origin of the NO source (endogenous or exogenous), as well as its intracellular levels, may act differently in the subcellular compartmentalization of *S*-nitrosylated proteins.

**FIGURE 5:**
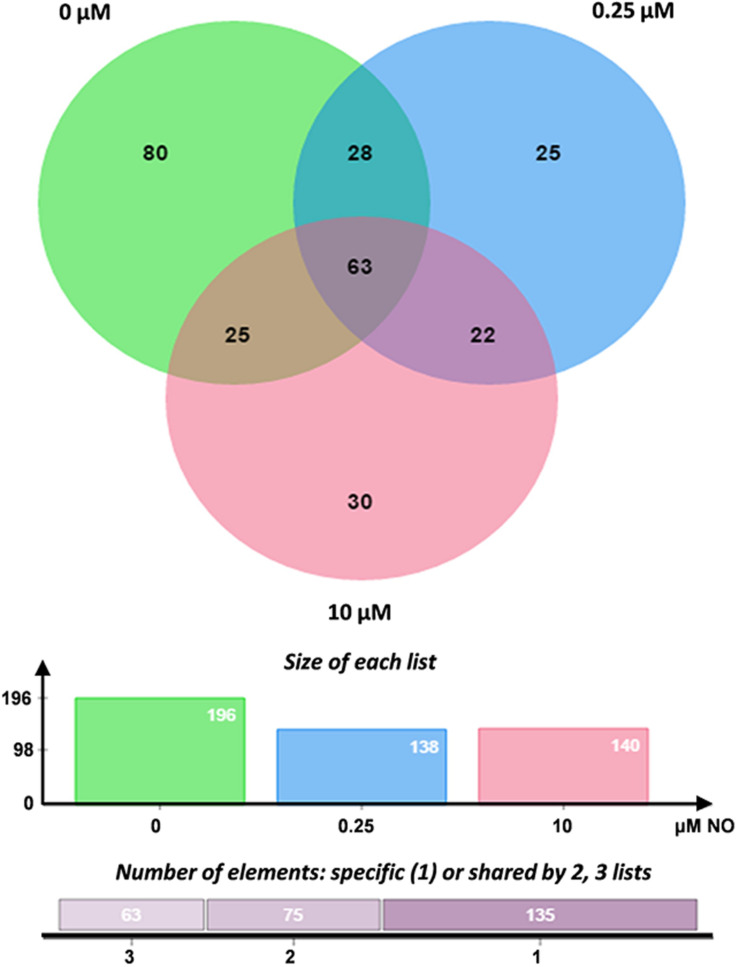
Venn diagram of *S*-nitrosylated proteins. Enriched proteins list generated by MaxQuant software were analyzed using Jvenn bioinformatics application (bioinfo.genotoul.fr/jvenn/index.html). The graphic shows the common and exclusive proteins in each sample treated with 0.25 and 10 μM NO. Number of proteins analyzed in each group.

The Venn diagram generated from these analyses represents the number of proteins identified in each sample, as well as the overlap of common elements. The complete list of the identified proteins is available in the Supplementary Materials. Overall, 25 and 30 *S*-nitrosylated proteins were detected exclusively in samples treated with 0.25 and 10 μM nitrite, respectively (Figure 5). The unique proteins detected in each sample are shown in Table 1. *S*-nitrosylated proteins identified from samples enriched with low NO concentrations exhibited the expected profile, with proteins related to cellular proliferation and several proteins related to metabolism and cell growth, while in samples enriched with high NO concentrations, we observed proteins involved in cell survival, possibly to repair damage caused by cellular redox imbalance.

**TABLE 1:** List of proteins exclusively found on *S*-nitroso proteome.

**ID**	**Uniprot**	**Protein**	*LFQ/100000
**Control group**			
PADG_01621	C1G3V5	Aspartate aminotransferase	3398.97
PADG_00873	C1FYJ7	Histone H3	2988.02
PADG_12076	A0A0A0HRG7	Actin	2666.26
PADG_07524	C1GJT8	Nucleoside diphosphate kinase	2185.69
PADG_12253	A0A0A0HUJ5	60S ribosomal protein L3	2098.36
PADG_01711	C1G445	Uncharacterized protein	1757.41
PADG_04056	C1G9X0	14-3-3 family protein épsilon	1586.01
PADG_05822	C1GEY6	Pyridoxine biosynthesis protein pyroA	1497.92
PADG_03856	C1G9C0	Ribosomal protein L15	1489.78
PADG_01314	C1G2Z8	YggS family pyridoxal phosphate enzyme	1327.09
PADG_07813	C1GKM7	ATP synthase subunit gamma	1314.40
PADG_02828	C1G6M3	Ribosomal protein	1285.74
PADG_07953	C1GKU6	Peptidyl-prolyl cis-trans isomerase	1270.76
PADG_02260	C1G294	Uncharacterized protein	1255.86
PADG_02555	C1G5V0	Uncharacterized protein	1177.62
PADG_04934	C1GBD3	Uncharacterized protein	1118.93
PADG_02249	C1G283	60S ribosomal protein L2	937.59
PADG_04099	C1GA13	Phosphoribosylaminoimidazolecarboxamide formyltransferase/IMP cyclohydrolase	935.75
PADG_05402	C1GDR6	Uncharacterized protein	900.69
PADG_04175	C1GA89	Inorganic pyrophosphatase	835.04
PADG_01387	C1G371	60S ribosomal protein L7	713.89
PADG_03466	C1G596	3-hydroxyisobutyrate dehydrogenase	710.63
PADG_05906	C1GF70	Histone H2A	705.23
PADG_05340	C1GDK4	Uncharacterized protein	691.36
PADG_00335	C1G0E5	40S ribosomal protein S14	676.05
PADG_06048	C1GFL2	40S ribosomal protein S27	639.72
PADG_04440	C1GB04	Uncharacterized protein	634.92
PADG_04516	C1GBZ4	Glutamate dehydrogenase	633.89
PADG_02805	C1G6K0	Isocitrate dehydrogenase [NAD] subunit, mitochondrial	614.55
PADG_00354	C1G0G4	40S ribosomal protein S17	612.26
PADG_00872	C1FYJ6	Histone H4	598.41
PADG_05517	C1GE31	Uncharacterized protein	555.52
PADG_01267	C1G2V1	40S ribosomal protein S11	502.35
PADG_04315	C1GAM9	40S ribosomal protein S24	498.56
PADG_05947	C1GFB1	Nicotinate-nucleotide pyrophosphorylase [carboxylating]	478.92
PADG_00937	C1FYR1	Uncharacterized protein	446.49
PADG_11379	A0A0A0HYV6	60S ribosomal protein L5	437.40
PADG_00615	C1G175	Proteasome subunit alpha type	398.50
PADG_00514	C1G0X4	60S ribosomal protein L16	364.94
PADG_03058	C1G7A3	Uncharacterized protein	330.52
PADG_08281	C1GLP0	Uncharacterized protein	323.09
PADG_07714	C1GKC8	Uncharacterized protein	304.08
PADG_08466	C1GMI0	Homogentisate 1,2-dioxygenase	270.73
PADG_08503	C1GML7	Phosphoenolpyruvate carboxykinase [ATP]	268.92
PADG_06490	C1GGQ3	Uncharacterized protein	267.37
PADG_04449	C1GB13	60S ribosomal protein L23	260.74
PADG_11128	A0A0A0HVT2	Uncharacterized protein	259.77
PADG_03825	C1G989	Uncharacterized protein	258.15
PADG_01455	C1G3D9	Uncharacterized protein	257.35
PADG_06726	C1GHJ0	60S ribosomal protein L17	234.71
PADG_01762	C1G496	Oxoglutarate dehydrogenase (Succinyl-transferring), E1 component	232.19
PADG_03194	C1G7N9	Uncharacterized protein	232.13
PADG_04307	C1GAM1	Uncharacterized protein	227.72
PADG_01706	C1G440	Uncharacterized protein	226.30
PADG_06978	C1GI92	Cytochrome c	221.65
PADG_06756	C1GHM0	Histidinol dehydrogenase	208.89
PADG_00824	C1FYE8	Uncharacterized protein	187.73
PADG_08465	C1GMH9	Fumarylacetoacetase	172.35
PADG_04034	C1G9U8	Chaperone DnaJ	170.91
PADG_06568	C1GH32	Uncharacterized protein	164.77
PADG_05893	C1GF57	Uncharacterized protein	160.31
PADG_07412	C1GJH6	Uncharacterized protein	144.24
PADG_02214	C1G248	4-aminobutyrate aminotransferase	133.92
PADG_05281	C1GDE5	Uncharacterized protein	132.09
PADG_01871	C1G4K5	Uncharacterized protein	119.42
PADG_05058	C1GBQ7	Chorismate mutase	110.77
PADG_07014	C1GIC8	Uncharacterized protein	107.76
PADG_08191	C1GMA5	Uncharacterized protein	97.84
PADG_08553	C1GMQ9	Uncharacterized protein	91.33
PADG_04452	C1GB16	Uncharacterized protein	89.27
PADG_03203	C1G7P8	Uncharacterized protein	87.67
PADG_01328	C1G312	Ornithine aminotransferase	75.75
PADG_04241	C1GAF5	Coatomer subunit alpha	71.14
PADG_04250	C1GAG4	Uncharacterized protein	65.74
PADG_00608	C1G168	Uncharacterized protein	57.93
PADG_00379	C1G0I9	Uncharacterized protein	53.39
PADG_01745	C1G479	Mannose-1-phosphate guanyltransferase	36.45
PADG_01083	C1FZ57	60S ribosomal protein L32	22.39
PADG_08177	C1GM91	Uncharacterized protein	18.08
PADG_02865	C1G6R0	Uncharacterized protein	3.77
**0.25 μM nitrite**			
PADG_02914	C1G6V9	Aminomethyltransferase	279.12
PADG_04848	C1GB47	60S ribosomal protein L8-B	213.26
PADG_04709	C1GCI7	Isocitrate lyase	175.59
TIF32	C1G9T0	Eukaryotic translation initiation factor 3 subunit A	158.94
PADG_06313	C1GG76	40S ribosomal protein S18	131.35
PADG_06546	C1GGV9	Puromycin-sensitive aminopeptidase	123.33
PADG_02764	C1G6F9	Uncharacterized protein	118.76
PADG_00451	C1G0R1	Glucose-6-phosphate isomerase	107.41
PADG_06805	C1GHR9	Acyl-CoA dehydrogenase	106.03
BNA5	C1G0F9	Kynureninase	100.76
PADG_02343	C1G2H7	Uncharacterized protein	86.52
PADG_02030	C1G514	Hsp90 co-chaperone Cdc37	74.29
PADG_04949	C1GBE8	Threonine-tRNA ligase	44.00
PADG_07435	C1GJJ9	Uncharacterized protein	35.30
PADG_03325	C1G820	Uncharacterized protein	31.16
PADG_04890	C1GB89	Uncharacterized protein	28.54
PADG_01755	C1G489	Superoxide dismutase	28.28
PADG_06144	C1GFV8	Saccharopine dehydrogenase [NAD(+), L-lysine-forming]	26.95
PADG_02895	C1G6U0	Heat shock protein	24.72
PADG_05338	C1GDK2	60S ribosomal protein L18	19.20
PADG_11413	A0A0A0HWY2	Uncharacterized protein	19.16
PADG_11981	A0A0A0HRM2	V-type proton ATPase catalytic subunit A	17.10
PADG_02637	C1G632	Uncharacterized protein	9.90
CPYA	C1GG77	Carboxypeptidase Y homolog A	9.30
**10 μM nitrite**			
PADG_08244	C1GLK3	60S acidic ribosomal protein P1	726.30
PADG_06838	C1GHV2	40S ribosomal protein S5	700.02
PADG_00446	C1G0Q6	Uncharacterized protein	459.28
PADG_05032	C1GBN1	Uncharacterized protein	458.46
PADG_01797	C1G4D1	Uncharacterized protein	279.19
PADG_00443	C1G0Q3	Dihydropteroate synthase	188.75
PADG_07627	C1GK41	Uncharacterized protein	180.29
PADG_07370	C1GJD4	Uncharacterized protein	172.67
PADG_00210	C1G020	Glycine dehydrogenase	156.30
PAD.G_04192	C1GAA6	Uncharacterized protein	122.04
PADG_01100	C1FZ74	Uracil phosphoribosyltransferase	120.38
PADG_06992	C1GIA6	GrpE protein homolog	114.80
PADG_00599	C1G159	26S protease regulatory subunit 6A	105.36
PADG_04100	C1GA14	Clathrin heavy chain	101.89
PADG_03984	C1G9P8	Glutamine-fructose-6-phosphate transaminase (Isomerizing)	100.42
PADG_02967	C1G712	Uncharacterized protein	85.66
PADG_12401	A0A0A0HTA9	Cytochrome c1, heme protein, mitochondrial	78.44
PADG_01228	C1G2R2	Uncharacterized protein	77.40
PADG_02683	C1G678	UV excision repair protein Rad23	53.88
PADG_03431	C1G561	Uncharacterized protein	43.02
PADG_06249	C1GG12	Glutaminyl-tRNA synthetase	41.72
PADG_05922	C1GF86	Cytosolic non-specific dipeptidase	40.61
PADG_00244	C1G054	Uncharacterized protein	31.44
PADG_07264	C1GJ28	Uncharacterized protein	30.53
PADG_03964	C1G9M8	Fumarylacetoacetate hydrolase domain-containing protein	26.75
PADG_04495	C1GBX3	Uncharacterized protein	20.40
PADG_04994	C1GBJ3	Uncharacterized protein	12.75
PADG_00183	C1FZZ3	Profilin	12.17
PADG_12214	A0A0A0HR13	Uncharacterized protein	8.59
PADG_03565	C1G8H9	Uncharacterized protein	1.80

*Label-free quantification.

Among exclusively *S*-nitrosylated proteins identified in both treatment groups, some are of interest and correlate with our previous results, such as superoxide dismutase (PADG_01755), which is involved in detoxifying the environment in response to oxidative stress and was exclusively found at low concentrations of NO. In this group, we identified several molecular chaperones, such as *Hsp90 co-chaperone Cdc37* (PADG_02030), *Hsp60* (PADG_08369), *Hsp72* (PADG_08118), *Hsp7* (PADG_00430), *Hsp90* (PADG_07715), and *Hsp98* (PADG_00765). Among those proteins, we can highlight the Hsp60 and Hsp90 proteins, which have been largely explored in fungal cells and are known to be essential for cell growth (Burnie et al., 2006), cell survival in the host, morphogenesis (Izacc et al., 2001; Thomaz et al., 2014), germination and conidiation (Lamoth et al., 2012; Tiwari et al., 2015), cell wall integrity (CWI) and drug resistance (Shapiro et al., 2009). We also identified the protein *eukaryotic translation initiation factor 3 subunit A* (PADG_04016) responsible for mitotic control, microtubule and cytoskeleton stabilization and mitotic control and the cell cycle. Several other proteins identified in the low concentrations of NO group were correlated with the process of metabolic cycle, cellular respiration, amino acid and carbohydrate biosynthesis, such as *heat shock protein* (PADG_02895), *saccharopine dehydrogenase* (PADG_06144), *puromycin-sensitive aminopeptidase* (PADG_06546), *acyl-CoA dehydrogenase* (PADG_06805), *glucose-6-phosphate isomerase* (PADG_00451), *kynureninase* (PADG_00349), *mitochondrial 2-methylisocitrate lyase* (PADG_04709), and *aminomethyltransferase* (PADG_02914). This profile is in keeping with the proliferative pattern previously found.

In the group treated with high levels of NO, in general, we identified proteins related to stress and ubiquitination, such as *26S protease regulatory subunit 6A* (PADG_00599) and *UV excision repair protein Rad23* (PADG_02683). Additionally, we identified proteins involved in protein degradation (*cytosolic nonspecific dipeptidase –* PADG_05922), amino acid degradation (*fumarylacetoacetate hydrolase domain-containing protein* – PADG_03964), and osmotic stress response (*Profilin –* PADG_00183). Interestingly, we also found the anti-apoptotic protein *glutaminyl-tRNA synthetase* (PADG_06249). Overall, proteins identified in this group display a survival profile when stressed with high concentrations of reactive nitrogen species.

It was demonstrated in *Candida albicans* that the response to nitrosative stress activates genes that regulate the expression of oxidative stress, such as catalase (Cat1), glutathione, NADPH oxidoreductase and dehydrogenase (Brown et al., 2014) elements that were identified on the proteomic analysis (Supplementary Table S1). In biological systems, cells have an antioxidant repertoire essential to ROS protection, which activates several effector molecules (Ribeiro et al., 2017).

### *S*-Nitrosylated Predicted Sites *in silico* Analysis

**FIGURE 6:**
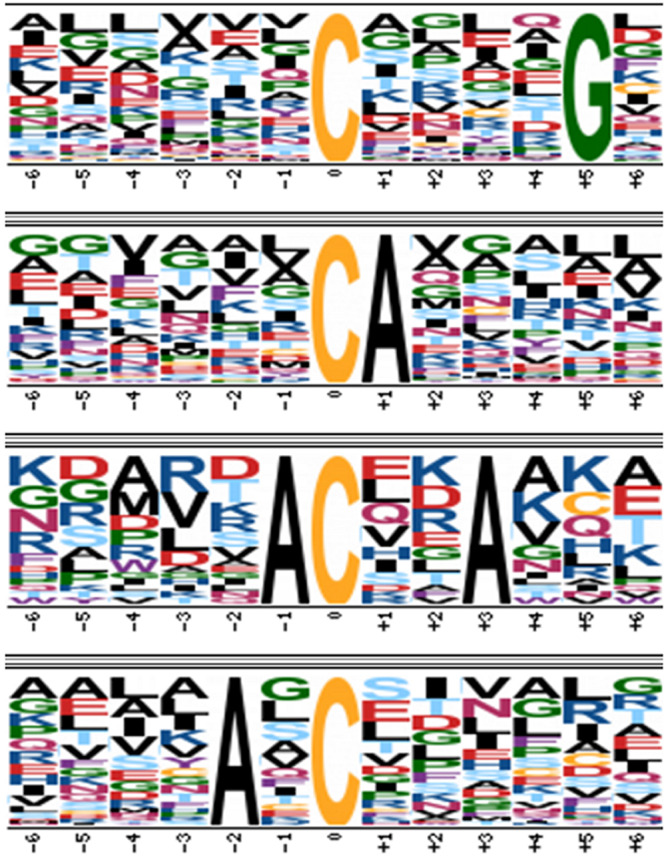
Motif analysis of *S*-nitrosylated proteins using the Motif-X algorithm. Motifs were identified using the Motif-X algorithm (http://motif-x.med.harvard.edu/motif-x.html). The background organism used was SGD Yeast Proteome and the central character was Cys with a significance of 10^6^.

Proteomic data were entered into the SNOSite search algorithm^2^ (Lee T.Y. et al., 2011) to identify predicted sites of *S*-nitrosylation (Supplementary Table S2). With this analysis, it was possible to observe that of the 275 proteins identified in Supplementary Table S1, 227 present predicted sites of *S*-nitrosylation, approximately 82% of all identifications. Despite such abundance, *S*-nitrosylation occurs only at select cysteine residues of proteins. The main *S*-nitrosylation motifs identified in this work were XX**CA**XX, XX**C**XXXX**G**X, XX**AC**XX**A**X, and XX**A**X**C**XX (Figure 6). In this study, we verified that the main characteristics of cysteine *S*-nitrosylation were present. Such selectivity depends on the target cysteines to be located in a region with a high presence of hydrophobic amino acids (e.g., Ala, Leu, and Phe) (Ischiropoulos and Gow, 2005; Möller et al., 2007). In addition, these cysteines have lower *pKa* and are surrounded by acidic and basic residues (Lys, Arg, His, Asp, and Glu) and bulky amino acid residues (e.g., Phe, Arg, and Leu) (Cheng et al., 2014). These charged amino acids would be more exposed and therefore would increase accessibility to SNO sites. These residues are also responsible for facilitating non-covalent interactions, which may promote S-nitrosylation (Hasan et al., 2019).

## Discussion

The capacity of *P. brasiliensis* to resist the deleterious effects of RNS/ROS has been explored as an important virulence mechanism, mainly concerning the host-pathogen interaction. In addition to phagocytosis, immune cells may also eliminate fungal cells using mechanisms beyond internalization. In this scenario, phagocytic cells produce high amounts of NO. Previous data demonstrated that high concentrations of NO lead to fungal death (Haniu et al., 2013; Parente et al., 2015; Conceição et al., 2019). On the other hand, low concentrations stimulate fungal proliferation (Haniu et al., 2013; Pengkit et al., 2016; Conceição et al., 2019). This evidence supports the role of RNS as messengers capable of modulating fungal responses through redox PTM and protein expression.

Signaling pathways control cellular survival, growth and function and are organized as a biochemical network that allows protein activity to specific responses. PTMs have a fundamental role in regulating a wide variety of mechanisms (Spickett et al., 2006). Intracellular redox status is directly involved in proliferation regulation and cell differentiation. Throughout evolution, microorganisms have developed the capacity to adapt and reproduce even when subjected to adverse conditions (e.g., nitrosative, oxidative, and osmotic stress). Changes associated with stress events are generally associated with the expression of proteins that prevent damage originating from those events (Tiwari et al., 2015).

The strong reactivity of side chain cysteine thiol groups has been widely investigated with proteins responsive to ROS and RNS. The cysteine redox state varies from +2 to +6, making this amino acid the most versatile in terms of redox regulation (Jacob et al., 2003). This reaction on organisms is highly labile and reversible. This reversibility and structural changes caused by oxidation allow signalization cascades to have a fine coordination of adaptive responses to react to endogenous and exogenous metabolic processes (Vázquez-Torres, 2012). Proteins *S*-nitrosylation is important to a considerable variety of mechanisms, from RNS detoxification in bacteria (Hess et al., 2005; Patra et al., 2016) and protozoa (Wang et al., 2014), to regulating cell cycle in fungi cells (Foster et al., 2009; Lee P.Y. et al., 2011; Majumdar et al., 2012) and regulating human diseases (Chung, 2015; Bradley and Steinert, 2016).

The protein *S*-nitrosylation (SNO-protein) can alter its conformation, enzymatic activity, protein-protein interaction, or cell location, consequently affecting the protein function. In the present study, we identified S-nitrosylated proteins in *Paracoccidioides brasiliensis* after treatment with different concentrations of NO. Currently, it is recognized that NO has a double-edged role in a dose-dependent manner (Ramesh et al., 2014), since in low concentrations its role is protective and acts an important signaling molecule, whereas in high concentrations it has important deleterious effects. Thus, *S*-nitrosylated proteins identified at low concentrations of NO, would represent proteins involved with fugal virulence. On the other hand, the analysis of *S*-nitrosylated proteins from *P. brasiliensis* exposed to high concentrations of NO could help explain the antifungal properties of RNS and consequently identify potential pharmacological targets.

We have presented different *S*-nitrosylated proteins identified in the fungus treated with low concentrations of NO and under high concentrations (Supplementary Table S1). Among the *S*-nitrosylated proteins found in the group treated with a high concentration of NO, we detected some potential pharmacological targets. These proteins are mainly involved with CWI, amino acid, and folic acid metabolisms.

Proteins involved in CWI pathways can be potentially used as antifungal drug targets (Valiante et al., 2015). We detected high levels of *S*-nitrosylation of the protein Arp2/3 complex subunit (PADG_05538) (Supplementary Table S1). Lee et al. (2016) demonstrated that the depletion of components of the Arp2/3 complex in *Candida albicans* increased the exposure of chitin and β-glucans to the cell wall, impairing CWI and reducing the fungal adhesion mediated by the small G-protein Rho1. The authors suggest that Arp2/3 inhibition could be an interesting strategy to block the formation of fungal biofilms (Lee et al., 2016).

Sba1 (PADG_05032) is a co-chaperone of Hsp90 and was detected *S*-nitrosylated only after treatment with NO (Supplementary Table S1). Hsp90 and its co-chaperones are essential for resistance to antifungals (Cowen, 2009). Sba1 deletion increased the susceptibility to azoles in *Fusarium verticillioides* and *Neurospora crassa*, indicating that the Hsp90-cochaperone is critical in adaptive responses to azoles and could be potential targets for developing of new antifungal agents (Gu et al., 2016).

Yeast cells can synthesize folate *de novo* via the folic acid synthesis pathway. In contrast, mammalian cells cannot synthesize folates and are totally dependent on exogenous folic acid (Raimondi et al., 2019). Dihydrofolate reductase (DHFR) and Serine Hydroxymethyltransferase (SHMT) (PADG_05277) are keys enzymes involved in this process, and its inhibition disrupts the biosynthesis of purine nucleotides, thymidine (precursor for DNA replication) and several amino acids (Djapa et al., 2006). SHMT produces tetrahydrofolate (THF) by an alternative route when the canonical production is blocked. SHMT inhibitors have been used as an alternative to fight malaria infection (Fernandes et al., 2018; Schwertz et al., 2018).

Mammals do not synthesize some amino acids and therefore depend on food sources for the acquisition of proteogenic amino acids (Amich and Bignell, 2016). Thus, amino acid metabolism pathways become attractive targets for antifungal therapies. We detected two proteins involved in cysteine biosynthesis, such as Cysteine Synthase (PADG_02726) and homocysteine S-methyltransferase (PADG_08328). Several studies have shown that using mutant lacking the cysteine synthase (to block the main pathway of cysteine biosynthesis) led to the absence of the cysteine and consequently limited the infection by *Aspergillus fumigatus* and the growth of *C. albicans* and *S. cerevisiae* (Bachhawat and Yadav, 2010; Amich et al., 2016). Thus, these studies suggested that Cysteine Synthase may be a new potential target for antifungal agents.

Another important protein identified in this study is the glyceraldehyde-3-phosphate dehydrogenase (GAPDH) (PADG_02411 – Supplementary Table S1). We identified GAPDH *S*-nitrosylated (SNO-GADPH) in all conditions analyzed (Supplementary Table S1). In GAPDH interactome analyzes was observed that SNO-GAPDH can interact with several *P. lutzii* proteins, such as acyl CoA dehydrogenase, argininosuccinate synthase, aldehydedehydrogenase, rad24, among others (Silva et al., 2019). GAPDH is well-known for its role in cytosolic glycolysis (Tristan et al., 2011). This redox PTM regulates both glycolytic enzyme activity (Padgett and Whorton, 1995) as well as its different “moonlighting” functions (Kornberg et al., 2010; Jia et al., 2012). SNO-GAPDH has transnitrosylase activity in the nucleus and mitochondria, playing an important role in the transcription regulation as well as apoptosis (Hara et al., 2005). Transnitrosylases are proteins that enzymatically transfer its NO moiety to another acceptor protein. In mammalian cells, SNO-GADPH can transnitrosylate the DNA-activating protein kinase (DNA-PK), a protein involved in DNA repair, leading to its activation (Kornberg et al., 2010; Jia et al., 2014). In addition, we identified the Thioredoxin (Trx – PADG_05504) and Protein disulfide-isomerase (Pdi1 – PADG_05822) *S*-nitrosylated in all evaluated conditions (Supplementary Table S1). These proteins control the degree of protein *S*-nitrosylation via thiol denitrosylation (Benhar et al., 2009). The identification of *S*-nitrosylated GAPDH, Trx and Pdi in *P. brasiliensis* indicates that transnitrosylation and denitrosylation, important redox signaling mechanisms, are present and preserved in this fungus.

Finally, this study is the first to undertake an analysis of *P. brasiliensis S*-nitrosylated proteins, highlighting its regulation and strategy to proliferate and survive in less favorable conditions. *S*-nitrosylation is an interesting redox PTM, which can modify the function of proteins and to control a large number of signaling pathways. The identification of such molecules could help to elucidate RNS antifungal properties, as well as determining potential molecular targets for the development of new drugs.

## Data Availability Statement

The raw data supporting the conclusions of this article will be made available by the authors, without undue reservation, to any qualified researcher.

## Author Contributions

MN, LI, and WB designed the experiments. MN, AC, DC, IC, JC, PC, LI, and BC performed the experiments. MN and AC analyzed the data. MN and WB wrote the manuscript. WB supervised this project and funded this project. All authors discussed the results and commented on the manuscript.

## Conflict of Interest

The authors declare that the research was conducted in the absence of any commercial or financial relationships that could be construed as a potential conflict of interest.

## Footnotes

1 http://www.UniProt.org/

2 http://csb.cse.yzu.edu.tw/SNOSite/

## Supplementary Material

FIGURE S1 Validation of LC-MS/MS data. **(A)** Peptides posterior error probability (PEP) score is calculated as the probability of false identification using peptide score and length. Longer peptides with high identification scores are automatically accepted. **(B)** Distribution and length of all identified peptides.
