# Supplementary figures and images for: Effect of Nitrosative Stress on the S-Nitroso-Proteome of Paracoccidioides brasiliensis
Source: Front Microbiol. 2020 Jun 4;11:1184. doi: 10.3389/fmicb.2020.01184 (PMC7287035; doi:10.3389/fmicb.2020.01184)

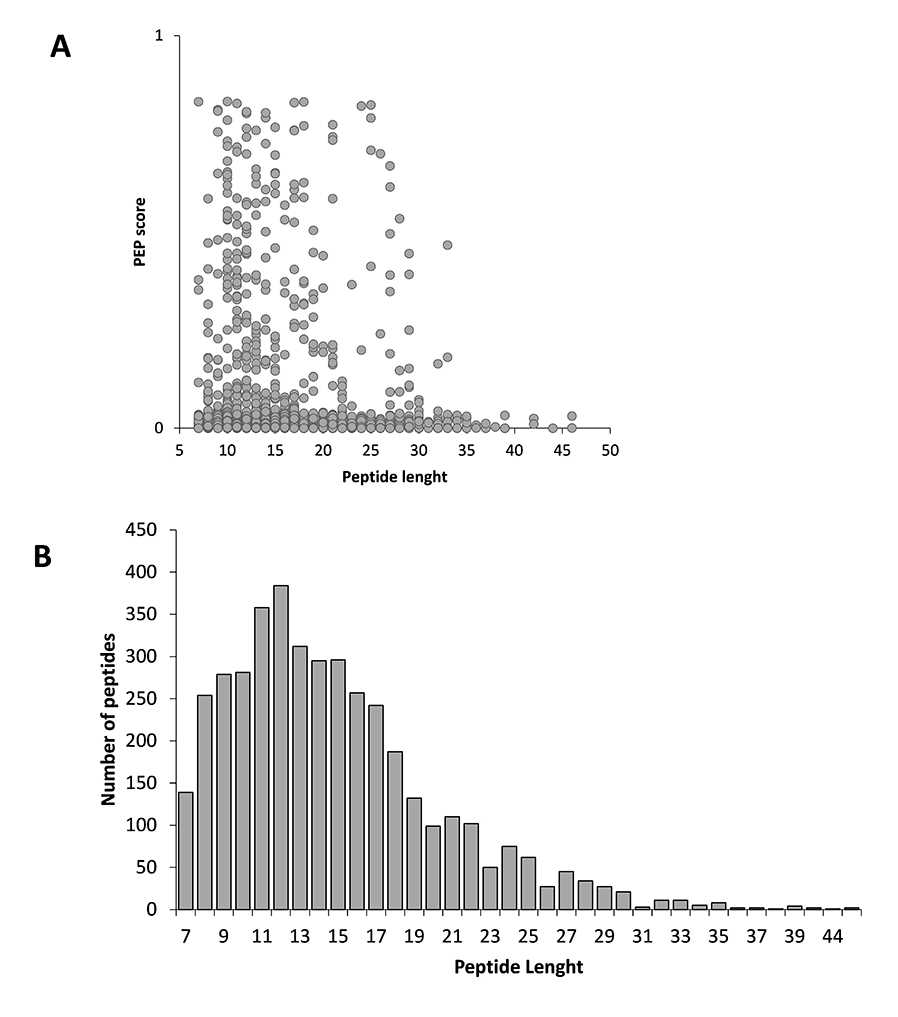

Supplement: FIGURE S1 — Validation of LC-MS/MS data. (A) Peptides posterior error probability (PEP) score is calculated as the probability of false identification using peptide score and length. Longer peptides with high identification scores are automatically accepted. (B) Distribution and length of all identified peptides. [file Image_1.TIF]
